# Validity and reliability of a Chinese version of the self‐evaluation of negative symptoms

**DOI:** 10.1002/brb3.2924

**Published:** 2023-03-13

**Authors:** Guangdong Chen, Jiayue Chen, Hongjun Tian, Chongguang Lin, Jingjing Zhu, Jing Ping, Langlang Chen, Chuanjun Zhuo, Deguo Jiang

**Affiliations:** ^1^ Department of Psychiatry Wenzhou Seventh Peoples Hospital Wenzhou China; ^2^ Department of Psychiatry Tianjin Medical University Affiliated of Tianjin Fourth Center Hospital, Nankai University Affiliated Tianjin Fourth Center Hospital, Tianjin Fourth Center Hospital Tianjin China; ^3^ Department of Psychiatry, Tianjin Anding Hospital Tianjin Mental Health Center of Tianjin Medical University Tianjin China

**Keywords:** negative symptoms, reliability, schizophrenia, SNS, validity

## Abstract

The negative symptoms of schizophrenia can be present at any clinical stage, but evaluating the negative symptoms always remains challenging. To screen the negative symptoms effectively, self‐evaluation should be introduced. To date, professional psychiatrists used almost all of the scales available to screen the negative symptoms but could not obtain an accurate outcome. At the same time, an advanced self‐assessment scale is needed to accompany the patients’ self‐feeling‐based treatment strategies to understand their feelings about their symptoms. Hence, Chinese self‐evaluation of negative symptoms (SNS) should be introduced in China. This study aims to examine the validity and reliability of the Chinese version of SNS. Two hundred patients with schizophrenia were included in this study and were evaluated entirely with the self‐assessed negative symptoms by the Chinese version. The correlation analysis was performed between SNS and the Scale for Assessment of Negative Symptoms (SANS) to assess the criterion validity of SNS for screening negative symptoms. Exploratory factor analysis was used to determine the constructive validity of the SNS. Two senior professional psychiatrists were involved in this assessment based on their clinical experience and capability to define the severity of the negative symptoms. Receiver operating characteristic curve (ROC) analysis was performed to assess the cutoff point of SNS. Cronbach's alpha coefficient and intraclass correlation (ICC) coefficient were used to determine the reliability of SNS. We have the following findings: The Chinese version of SNS demonstrated a significant correlation with the SANS (*r* = .774, *p* < .05). Exploratory factor analysis demonstrated that the factor loading varies from .442 to .788. ROC analysis demonstrated that at SNS ≥ 8, the patients demonstrated a mild severity of negative symptoms, and at SNS ≥ 15, the patients demonstrated a severe severity of negative symptoms. Subsequently, 9 < SNS < 14 was defined as a moderate severity of negative symptoms. The Cronbach's alpha and ICC coefficients of the Chinese version SNS were .877 and .774, respectively. Our results showed that the acceptable validity and reliability of the Chinese version of SNS confirmed that SNS is an ideal tool for self‐assessment of the negative symptoms in patients with schizophrenia.

## Graphical abstract

Chinese version of the self‐evaluation of negative symptoms is an ideal tool for patients with schizophrenia to self‐assessment their negative symptoms. Chinese version of the self‐evaluation of negative symptoms demonstrated satisfactory psychometric properties, can providE more useful information for making tailored treatment strategy.

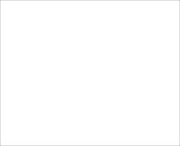

## 1 INTRODUCTION

The negative symptoms of schizophrenia include volitional (motivational) impairment manifesting as avolition, anhedonia, social withdrawal, and emotional disorders such as alogia and affective flattening (Galderisi et al., 2013; Millan et al., 2014). Previous cross‐sectional studies have reported that the prevalence of negative symptoms in patients with schizophrenia is approximately 50%–60% having at least one negative symptom showing moderate severity and about 10%–30% experiencing two or more negative symptoms (Galderisi et al., 2018). Mounting previous studies reported that negative symptoms, such as motivation, a flattening of emotional responses, a reduction in speech and activity, and social withdrawal, contribute to much of the disability associated with schizophrenia and associated with poor psychosocial functioning, subsequently causing a reduced likelihood of remission (Dominguez et al., 2010; Foussias et al., 2011; Hunter & Barry, 2012; McGurk et al., 2000; Rabinowitz et al., 2013).

According to previous reports, negative symptoms can be divided into primary negative symptoms and secondary negative symptoms (Kirkpatrick, 2014a, 2014b; Mosolov & Yaltonskaya, 2022). From the diagnostic point of view, it is important to differentiate between primary negative and negative symptoms (Mosolov & Yaltonskaya, 2022). Primary negative symptoms are regarded as an integral dimension of schizophrenia; the primary negative symptoms are intrinsic to the underlying pathophysiology of schizophrenia and can be observed at the initial stage of schizophrenia and continue as the illness progresses daily (Austin et al., 2015; Jann, 2004; Rector et al., 2005). Secondary negative symptoms result from positive symptoms, comorbid depression, side effects of antipsychotics, substance abuse, or social isolation. If secondary negative symptoms overlap with primary negative symptoms, it can create a false clinical impression of worsening deficit symptoms and disease progression, which leads to the choice of incorrect therapeutic strategy with excessive dopamine blocker loading (Barlati et al., 2022; Farreny et al., 2018). Secondary symptoms are related to psychiatric or medical comorbidities, adverse effects of treatment, or environmental factors (Gupta et al., 2021; Kirschner et al., 2017). Previous studies reported that negative symptoms of schizophrenia usually had five domains: (1) anhedonia—inability to feel pleasure; (2) avolition (apathy)—lack of energy and initiative, and loss of interest in usual activity; (3) social withdrawal—disturbed social activity and avoidance of interpersonal contacts; (4) alogia—negative cognitive disorder, narrowing of speech range, and poverty of content of speech; (5) emotional (affective) flattening or blunting and reduced emotional response to stimuli (Blanchard & Cohen, 2006; Demyttenaere et al., 2021; Galderisi et al., 2013;). Currently, these five domains of negative symptoms were acquired supporting by a meta‐analysis (Demyttenaere et al., 2021); this information indicated that in clinical practice, we should carefully assess the negative symptoms; this careful assessment will provide more useful information for us to make tailored treatment strategy.

More awfully, there are relatively fewer articles focusing on standardized tools such as the Scale for Assessment of Negative Symptoms (SANS) (Andreasen, 1989), the negative symptoms’ subscale within the positive and negative syndrome scale (PANSS) (Kay et al., 1987), and the Brief Negative Symptom Scale (Kirkpatrick et al., 2011) that are used in the clinical practices to assess the negative symptoms in patients with schizophrenia. However, the SANS and negative symptoms subscale within the PANSS are the observer assessment scales. However, they can be subjected to a quantified assessment to check the severity of negative symptoms in patients with schizophrenia (Huber & Gross, 1989; Klosterkötter, 1992; Schultze‐Lutter et al., 2010). Until now, several scales have been developed to assess the subjective experience of negative symptoms in patients with schizophrenia, including the Subjective Experience of Deficits in Schizophrenia (Liddle & Barnes, 1988), Subjective Deficit Syndrome Scale (Jaeger et al., 1990), and Motivation and Pleasure Scale Self‐Report (MAPSR) (Selten et al., 1993). These scales have some limitations. For instance, the older scales are not self‐assessment, and the MAPRS fails to examine all the five dimensions (Llerena et al., 2013).

Self‐evaluation of negative symptoms (SNS) is a potential self‐assessment scale that tools the identification of negative symptoms of schizophrenia, which was initially developed by Dollfus et al. (2016) and was commonly used in clinical practice due to its efficiency. The SNS is a self‐assessment scale comprising 20 items, including the five dimensions of negative symptoms of schizophrenia. Each item in SNS gives a score of either 0 (*strongly agree*), 1 (*somewhat agree*), or 2 (*strongly disagree*) (Dollfus et al., 2016). The total score ranges from 0 to 40. The higher the score, the more severe the negative symptoms, that is, the five dimensions of SNS, including motivation, sociality, alogia, anhedonia, and diminished emotional range. Using SNS, the doctor can fully understand the five dimensions of negative symptoms in patients with schizophrenia; thereby, the doctor can gain more information to initiate a better tailored treatment strategy for patients with schizophrenia (Dollfus et al., 2016; Polat et al., 2022). Although SNS is a commonly used tool globally (García‐Álvarez et al., 2022; Hajj et al., 2020; Montvidas et al., 2020; Polat et al., 2022; Rodrguez‐Testal et al., 2019), to our knowledge, this Chinese version of SNS lacks the scope for better schizophrenia treatment. Hence, in this study, we aimed to examine the reliability and validity of the Chinese version of SNS in a group of Chinese‐speaking patients with schizophrenia.

## 2 METHODS>

### 2.1 Participants

The study included 200 Chinese‐speaking patients with schizophrenia who were hospitalized and interviewed using the Structured Clinical Interview for DSM‐IV Axis I Disorders (SCID‐I) for a lifetime diagnosis of schizophrenia according to the diagnostic interview by two senior psychiatrists used SCI‐D (First et al., 2012). All the participants were accepted to receive the antipsychotic agent's treatment. Further, care was taken while excluding the participants, considering the past event of coma for more than 5 min due to any reason, having a history of neurological disorders, severe physical traumas, history of substance abuse, intellectual disability, and so on. Patients or their guardians signed the written informed consent. The Ethics Committee of Tianjin Fourth Center Hospital approved this study (2018K0078).

### 2.2 Introduction of SNS

The SNS is a self‐assessment scale comprising 20 items, including the five dimensions of negative symptoms of schizophrenia. Each item in SNS gives a score of either 0 (*strongly agree*), 1 (*somewhat agree*), or 2 (*strongly disagree*) (Dollfus et al., 2016). The total score ranges from 0 to 40. The 20 items of SNS are as follows: (1) I prefer to be alone in my corner; (2) I am better off alone, because I feel uncomfortable when anyone is near me; (3) I am not interested in going out with friends or family; (4) I do not mainly try to contact and meet friends (letters, telephone, text messaging, etc.); (5) People say I am not sad or happy and that I am not often angry; (6) There are many happy or sad things in life but I do not feel concerned by them; (7) Watching a sad or happy film, or reading or listening to a sad or happy story does not especially make me want to cry or laugh; (8) It is difficult for people to know how I feel; (9) I do not have as much to talk about as most people; (10) I find it 10 times harder to talk than most people do; (11) People often say that I do not talk much; (12) With friends and family, I want to talk about things but it does not come out; (13) I find it difficult to meet the objectives I set myself; (14) It is hard to stick to doing things on an everyday regular basis; (15) There are many things I do not do through lack of motivation or because I do not feel like it; (16) I know there are things I must do (get up or wash myself for example) but I have no energy; (17) I do not take any great pleasure in talking to people; (18) I find it hard to take pleasure even when doing things I have chosen to do; (19) When I imagine doing one thing or another, I do not feel any particular pleasure in the idea; (20) I am not interested in having sex.

### 2.3 The Chinese version of the SNS translation and re‐translation procedure

First, SNS was translated into Chinese version by two senior Chinese‐speaking psychiatrists, and another 20 psychiatrists further checked it to assure its readability and understandability. The second aspect was to retranslate this Chinese version of SNS into the English version, which was translated by Mr. S. P. Chou, who was blinded to the original English SNS version. Finally, the Chinese version of SNS was decided on and approved by 22 Chinese psychiatrists and Mr. S. P. Chou.

### 2.4 Symptom assessment

All of the 200 patients with schizophrenia completed the self‐evaluated SNS. After the completion of self‐evaluated SNS, 20 senior professional psychiatrists completed an assessment of the Scale for the Assessment of Negative Symptoms (SANS). They defined the severity of negative symptoms for all 200 patients with schizophrenia by their clinical experience and the total scores of SANS and Global Clinical Impressions (Luther et al., 2018; Silberstein & Harvey, 2019; Velligan et al., 2020; Wójciak & Rybakowski, 2018).

### 2.5 Statistical analyses

Convergent validity was calculated by applying the Spearman correlation coefficient to compare the SNS scores and the corresponding scores of SANS. Confirmatory factor analysis was also used to assess the constructive validity of the SNS (Dollfus et al., 2022). Senior psychiatrists defined the severity of the negative symptoms according to their clinical experiences. Receiver operating characteristic curve (ROC) analysis was performed to determine the cutoff point of SNS (Zanin et al., 2022). Cronbach's alpha coefficient and intraclass correlation (ICC) coefficient were used to assess the reliability of SNS (Carlson et al., 2021).

## 3 RESULTS

### 3.1 Demographic and clinical characteristics

The sociodemographic and clinical information of all the 200 Chinese patients with chronic schizophrenia is listed in Table 1. Initially, the chlorpromazine equivalent was used to maintain the dosage consistency of antipsychotic agents in the patients accepted for this procedure.

**TABLE 1:** Demographic characteristics of patients with schizophrenia (*N* = 200) who participated in the present study

Variables	Patients with schizophrenia (*N* = 200)
Age (years)	35.2 ± 3.9
Gender (male/female)	93/107
Onset age (years)	21.5 ± 3.9
Illness duration (years)	5.8 ± 2.1
Cumulative antipsychotic agents’ dosage (mg) Chlorpromazine equivalent	3,296,845.0 ± 284,036.5

### 3.2 Validity

#### 3.2.1 Constructive validity

The confirmatory factor analysis was performed with Robust Diagonally Weighted Least Squares (RDWLS) estimation on the asymptotic covariance matrix. The exploratory factor analysis (EFA) found adequate values in the Kaiser‐Meyer‐Olkin (KMO) (.900, 95% confidence interval [CI] = .884, .903) and Bartlett's Sphericity (*χ* ^2^ (196) = 18,054.450, *p* < .001) tests. Parallel analyses recommended a one‐factor solution; however, Schwarz's Bayesian information criterion and scree plot initially suggested a five‐factor solution. These five factors coincide with the dimensions proposed by the authors of the scale and explain 62% of the variance. The factor's loading is shown in Table 2. The correlations between factors are demonstrated in Table 3. The five factors extracted from EFA (Model 1), the two factors proposed by the scale's authors (Model 2), and a model including five first‐order factors and one second‐order factor (Model 3) would allow the SNS scale to be used by adding up its items to get a total score, and a unidimensional model following the recommendation of the parallel analyses. All the models had adequate fit indicators. In addition, to check whether the five‐factor structure would be appropriate for participants with psychosis (schizophrenia), Model 3 was tested with subjects with a score more than or equal to the 90th percentile on the SNS scale. The goodness‐of‐fit indicators were adequate: Satorra–Bentler *χ* ^2^ = 258.44 (*df* = 165), root mean square error of approximation (RMSEA) = .034 (CI: .025, .047), comparative fit index (CFI) = .935, non‐normed fit index (NNFI) = .904, and standardized root mean square residual (SRMR) = .076 (Table 2).

**TABLE 2:** Exploratory factor analysis‐rotated factor matrix loadings

Items	DER	AN	AL	AV	SW
1	−.058	−.129	.390	.583	.700
2	.284	.233	.046	.028	.713
3	.122	.230	−.010	.019	.369
4	.063	.246	−.031	−.008	.403
5	.397	.033	.201	−.004	−.117
6	.603	.032	−.008	.084	.050
7	.478	.069	−.035	−.039	−.097
8	.399	−.139	.256	.110	.139
9	.233	−.308	.382	.097	.149
10	−.201	−.018	.820	.059	.039
11	.040	−.030	.778	−.075	−.025
12	.005	−.080	.320	.285	.118
13	−.094	−.078	.086	.658	−.010
14	.023	.066	−.025	.639	−.025
15	.069	−.084	.045	.777	.704
16	.099	.054	−.009	.468	.045
17	.089	.456	.216	−.008	.303
18	.085	.528	.119	.256	.048
19	.192	.563	.017	.120	−.004
20	−.019	.303	.118	−.156	.060

Abbreviations: AL, alogia; AN, anhedonia; AV, avolition; DER, diminished emotional range; SW, social withdrawal.

**TABLE 3:** Fit indices of the SNS scale

Model	*χ* ^2^ Satorra–Bentler	*df*	comparative fit index (CFI)	non‐normed fit index (NNFI)	standardized root mean square residual (SRMR)	root mean square error of approximation (RMSEA) [90% CI]	Akaike information criterion (AIC)
Model 1	589.597	112	.902	.923	.052	.039 [.032–.051]	633.360
Model 2	557.420	120	.942	.963	.075	.069 [.582–.729]	576.290
Model 3	583.174	135	.930	.974	.049	.042 [.040–.423]	603.25
Model 4	569.334	146	.906	.921	.079	.075 [.069–.082]	587.456

*Note*. Model 1: Five factors found from exploratory factor analysis (EFA); Model 2: Two factors proposed by the scale's authors; Model 3: One second‐order factor and five first‐order factors found by EFA; Model 4: unidimensional model.

#### 3.2.2 Criterion validity

The correlation coefficient of SNS and SANS total scores was .774 (95% CI: .750–.801), and the correlation coefficient of the five subscales of SNS and corresponding five dimensions scores of SANS varied from .799 to .886 (95% CI ranges from .708–.813 to .805–.903; Table 4). The correlation coefficient of SNS and GCI was .800. These data demonstrated that the SNS had ideal criteria validity for self‐assessment.

**TABLE 4:** Inner correlation of the five dimensions of SNS

Dimensions	Alogia	Avolition	Anhedonia	Social withdrawal	Diminished emotional range
Alogia	1	.540	.625	.515	.807
Avolition	.562	1	.762	.601	.625
Anhedonia	.624	.635	1	.752	.774
Social withdrawal	.485	.584	.744	1	.695
Diminished emotional range	.782	.715	.735	.695	1

### 3.3 Reliability

Internal consistency of the 20 items of SNS was calculated using Cronbach's alpha coefficient. Our data demonstrated that Cronbach's alpha for the total score was .967. The internal consistency coefficient of the five subscales of SNS varied from .485 to 1; the detailed information is presented in Table 3. In addition, Cronbach's alpha coefficient and ICC coefficient of Chinese version of SNS were .877 and .774, respectively. The coefficient of the inner correlation of the five dimensions of SNS varies from .485 to 1 (Table 5).

**TABLE 5:** Correlation between SNS and SANS

Correlation between SNS and SANS	Co‐efficient	95% CIs
Alogia in SNS correlated with Alogia in SANS	.799	.708–.813
Avolition in SNS correlated with Avolition Apathy in SANS	.831	.755–.890
Anhedonia in SNS correlated with Anhedonia Asociality in SANS	.854	.700–.911
Social withdrawal in SNS correlated with Anhedonia Asociality in SANS	.902	.884–.952
Diminished emotional range in SNS correlated with Affective Blunting in SANS	.899	.854–.921
Attention subscale in a subscale of “Disorganization” dimension	.996	.925–1.00

### 3.4 ROC analysis

Our data demonstrated that SNS's total score cutoff value was 8. It can discriminate against schizophrenia patients with mild negative symptoms. The area under ROC was .894 (95% CI: .705–.873, *p* *<* .001), sensitivity was .887, and specificity was .960. When the cutoff value is ≥15, the SNS can discriminate against schizophrenia patients with severe negative symptoms. The area under ROC was .900 (95% CI: .852–1, *p* < .001), sensitivity was .911, and specificity was .888. Subsequently, 9 < SNA < 14 was defined as a moderate severity of negative symptoms (Table 6).

**TABLE 6:** Sensitivity and specificity of SNS for different threshold values demonstrated by ROC

Cutoff point	Sensitivity	Specificity
6	.989	.526
7	.980	.601
8	.920	.892
9	.906	.897
10	.900	.902
11	.899	.926
12	.875	.938
13	.870	.956
14	.860	.969
15	.855	.968
16	.653	.987

## 4 DISCUSSION

In this study, we examined the validity and reliability of the Chinese version of SNS as a self‐assessment scale on our data and also demonstrated good validity and reliability in evaluating the negative symptoms of Chinese patients with schizophrenia. Hence, the Chinese version of SNS is a useful tool for schizophrenia patients to assess their negative symptoms from the five dimensions. More notably, through the Chinese version of SNS, doctors can also acquire additional useful information from the patients’ experience, which subsequently can help provide a more appropriate tailored treatment strategy, following the guidelines based on patients’ self‐feeling to provide novel treatment strategies.

In a process to empower schizophrenia patients’ treatment that aims to improve the prognostic of these patients, self‐evaluation tools are necessary so that patients themselves can precisely describe their self‐experiencing of negative symptoms in different dimensions. Based on this self‐experience of negative symptoms, doctors can tailor treatment strategies that consider the patient's experience. This category‐tailored treatment strategy not only considers the doctor's clinical experiences but also considers the patients’ self‐feeling, which will favor the patients more than the treatment strategy that is only based on the doctor's clinical experience, and subsequently, it can help improve the prognostic of the patients with schizophrenia.

Given the benefits of patients' self‐feeling‐based treatment strategies, future clinical practices should emphasize the improvement of the negative symptoms in patients with schizophrenia (Correll, 2020; Harris & Panozzo, 2019; Morin & Franck, 2017). Forgoing these procedures, it became necessary to have a standard self‐evaluate instrument with satisfactory psychometric features for countries and ethnicities with different languages. It should be specified in such a manner that no self‐evaluation tool to evaluate the negative symptoms identified in schizophrenia studies has been translated and validated into Chinese language. Therefore, in the process to investigate the self‐assessment of patients with schizophrenia in the five negative domains of avolition, anhedonia, alogia, social withdrawal, and diminished emotional range, SNS can be considered an ideal valuable self‐evaluation tool for Chinese patients with schizophrenia.

In a follow‐up study, the characteristics of negative symptoms in the patients with the first episode of schizophrenia with the SANS were examined, and found that the negative symptoms were associated with the duration of the disease that changes as the disease progresses (Ergül & Üçok, 2015). More importantly, in the present study, our data demonstrated that the Chinese version of SNS can efficiently discriminate the severity of the negative symptoms of schizophrenia; the correlation coefficients between the Chinese version of SNS and the Chinese version of SANS in five dimensions are above .774, and these data confirmed that SNS, as a self‐assessment scale, had the equality power in discriminating the severity of negative symptoms as the observer‐assessment scale (SANS). The cutoff values were 8, 9–14, and 15, corresponding to the mild, moderate, and severe severity of the negative symptoms; the area under the ROCs converged to support the ideal sensitivity and specificity of the Chinese version of SNS. In summary, the Chinese version of SNS had the ability for the self‐assessment of the negative symptoms of schizophrenia by the patients.

## 5 LIMITATIONS

There are some limitations in our study. First, test–retest reliability was not examined in this study; future research is required to assess the reliability of the Chinese version of SNS over time. Second, a relatively small number of patients participated in the study; in the future study, we will enroll more participants to conduct the study to modify the Chinese version of SNS further. Third, in this study, we did not test the difference in SNS in the female and male patients; hence, we can avoid the influence of gender on SNS in the future large sample study, and we will make up for this limitation.

## 6 CONCLUSION

The Chinese version of SNS demonstrated satisfactory psychometric properties, supporting that this version is a useful scale for self‐assessment of the negative symptoms in patients with schizophrenia, and can provide more useful information for making tailored treatment strategies.

## AUTHOR CONTRIBUTIONS

**Guangdong Chen, Jiayue Chen, and Hongjun Tian** conceptualized the idea of the study, designed the methodology, provided software, performed analysis and investigation, and wrote the original draft. **Langlang Chen, Chongguang Lin, and Jingjing Zhu** provided software, performed analysis, and reviewed and edited the manuscript. **Jing Ping, Chuanjun Zhuo**, and **Deguo Jiang** conceptualized the idea of the study and performed supervision.

## CONFLICT OF INTEREST STATEMENT

The authors declare no conflicts of interest.

### PEER REVIEW

The peer review history for this article is available at https://publons.com/publon/10.1002/brb3.2924.

## Data Availability

The original contributions presented in the study are included in the article; further inquiries can be directed to the corresponding authors.
